# Central auditory processing and migraine: a controlled study

**DOI:** 10.1186/1129-2377-15-72

**Published:** 2014-11-08

**Authors:** Larissa Mendonça Agessi, Thaís Rodrigues Villa, Karin Ziliotto Dias, Deusvenir de Souza Carvalho, Liliane Desgualdo Pereira

**Affiliations:** 1 Division of Investigation and Treatment of Headaches (DITH), Neurology and Neurosurgery Department, Federal University of São Paulo, UNIFESP, São Paulo, SP, Brazil; 2 Speech Pathology and Audiology Department, Federal University of São Paulo, UNIFESP, São Paulo, SP, Brazil

**Keywords:** Migraine, Central auditory processing, Memory, Attention, Auditory cortex

## Abstract

**Background:**

This study aimed to verify and compare central auditory processing (CAP) performance in migraine with and without aura patients and healthy controls.

**Methods:**

Forty-one volunteers of both genders, aged between 18 and 40 years, diagnosed with migraine with and without aura by the criteria of “The International Classification of Headache Disorders” (ICDH-3 beta) and a control group of the same age range and with no headache history, were included. Gaps-in-noise (GIN), Duration Pattern test (DPT) and Dichotic Digits Test (DDT) tests were used to assess central auditory processing performance.

**Results:**

The volunteers were divided into 3 groups: Migraine with aura (11), migraine without aura (15), and control group (15), matched by age and schooling. Subjects with aura and without aura performed significantly worse in GIN test for right ear (p = .006), for left ear (p = .005) and for DPT test (p < .001) when compared with controls without headache, however no significant differences were found in the DDT test for the right ear (p = .362) and for the left ear (p = .190).

**Conclusions:**

Subjects with migraine performed worsened in auditory gap detection, in the discrimination of short and long duration. They also presented impairment in the physiological mechanism of temporal processing, especially in temporal resolution and temporal ordering when compared with controls. Migraine could be related to an impaired central auditory processing.

**Clinical trial registration:**

Research Ethics Committee (CEP 0480.10) – UNIFESP

## Background

Migraine is a neurological disease associated with an altered cortical excitability level [1]. Neurophysiological reports have shown that migraine is associated with abnormal excitability in visual, somatosensory and motor cortices [2-5]. The prevalence of migraine in Brazil is 15.2%, and migraine is more frequent in women and in individuals with higher education [6].

There is evidence that migraine patients could present cognitive deficits, being the affected functions memory, processing information speed and attention [7]. The cognitive dysfunction is present in the interictal phase and during migraine attacks [8]. Neuropsychological exams combined with imaging methods showed the existence of cortical dysfunction associated with impaired cognition [7].

The auditory ability to recognize, identify, and sequence sounds involves perceptual and cognitive processes [9].

Central auditory perception (CAP) can be assessed by behavioural tests, which demonstrate good correlations with electrophysiological measures [10].

Previous research has reported abnormalities in the auditory brainstem response (ABR). These results were an indicator of impending auditory malfunction in migraine and disruption of central sensory processing mechanisms could be one of the mechanisms predisposing a migraine sufferer to the increase in sensitive to sound, resulting in phonophobia [11]. In line with these symptoms, long-term and short-term habituation of auditory event-related potentials have revealed abnormal auditory processing in migraine, both interictally and during attacks [12].

In previous studies the multifeature sound mismatch negativity (MMN) was evaluated in children and adults with migraine and women eith menstrualy-related migraine [13-15]. In the pediatric population, a decrease in M150 amplitude during the migraine attack was observed [13]. In adults with migraine with aura, an increased N1 was reported, suggesting a hypoactivity of automatic cortical processes [14]. Interestingly, in the group of patients with menstrualy-related migraine was observed normal auditory sensory processing but increased automatic attention orienting processes to auditory changes [15].

The definition of CAP is based on auditory functions [16]. CAP involves a large number of skills which are highly dependent on a set integrity of the auditory pathways, from the outer ear to the auditory cortex. These skills include auditory selective attention, sound detection, localization, discrimination of isolated and sequential sounds, as well as speech recognition, auditory comprehension and memory [16-18].

CAP is an indispensable tool for the investigation of the function of the central nervous system [19]. The impairment of auditory processing could cause difficulties in complex listening situations, such as understanding speech in back-ground noise, rapid or degraded speech, and problems with comprehending verbal instructions [18,20].

A recent study that evaluated children with primary headache showed a deficit of auditory processing in noisy background compared to control cases [21].

The present study compared CAP performance between patients with migraine with and without aura and healthy controls.

## Methods

### Participants

Adults from both genders, aged between 18 and 40 years, were recruited via an advertisement in local community (São Paulo city). All the participants were assessed by the same headache specialist, at out-patient Division of Investigation and Treatment of Headaches (DITH), at the Federal University of Sao Paulo (UNIFESP). They were divided into two groups according to the International Headache Classification (ICDH-3 beta) criteria [22], confirmed by a completed 30-day filled headache diary: migraine with aura group (MA) and migraine without aura group (MwA). The control group (CG) had no previous history of headaches in the year prior to the study, and no migraine headaches in their lifetime.

Volunteers were excluded if their medical history and/or neurological exam suggested other neurological, psychiatric and systemic disorders, head trauma, hearing loss, ear trauma surgery or history of ototoxicity, use of medication (including migraine prophylaxis), occupational noise exposure, and the history of drug abuse or dependency, including that related to alcohol consumption and cigarette smoking. However, participants who used medication for acute attacks were allowed in the experiment.

Both migraineurs groups and controls were matched by age and schooling (counted in years from the elementary school).

The study was approved by the local ethical committee (trial register: CEP 0480.10 UNIFESP) and all subjects signed an informed consent form before participation. The data was collected between June 2011 and April 2012.

Gaps-in-Noise (GIN) test, duration pattern test (DPT) and dichotic digits test (DDT) were used to assess central auditory processing. All the procedures lasted 50 minutes and the tests were done in the same day. By the time the participants with migraine underwent the auditory processing assessment they had been symptom-free for at least of 3 days, according to the headache diary. The migraineurs had no attacks during and after the experiment.

The tests used to evaluate the central auditory processing were recorded on a compact disc, played on a CD player and passed through a GSI 61 audiometer to TDH-50 matched earphones.

#### Gaps-in-noise (GIN) test

The GIN [23] is composed of a series of 6-second segments of broadband noise containing 0 to 3 silent intervals or gaps per noise segment. The interstimulus interval between successive noise segments lasts 5 seconds and the gap durations presented are 2, 3, 4, 5, 6, 8, 10, 12, 15 and 20 miliseconds. The number of gaps and the duration per segment is varied. Ten practice items precede the test. There were six segments for each gap duration on each list, and two lists were used. The subjects were instructed to raise their thumb as soon as they heard a gap. An error was counted if a subject failed to raise his/her thumb. If a subject raised his/her thumb, without a gap having occurred, a false-positive was counted. To establish shortest gap durations, subjects needed to score a minimum of four out six at one gap duration.

#### Duration Pattern Test (DPT)

The DPT test [24] is a sequence of three 1,000 Hz tones, each one had three tones with different tone duration. The tone duration varies, one lasting 250 milliseconds, referred to as short, and another of 500 milliseconds, referred to as long. The interstimulus intervals were maintained at 300 milliseconds between the sequential tones, and the rise-descent time was kept at 10 msec. The test sequences were presented at an intensity of 30 dBSL based on the auditory thresholds in the frequencies between 500 and 2,000 Hz in both ears, with TDH-39 earphones. The patients were instructed to repeat the three-tone sequence in the same order he/she had heard it. Before testing, five practice items are provided to each subject to ensure their understanding of the task.

#### Dichotic digit test

The dichotic digit Portuguese version [25] test is a list of dissylabic words (numbers) spoken simultaneously and dichotically. Subjects are asked to listen attentively and repeat. There are twelve lists divided into pairs, each one containing twenty numbers.

### Data analysis

For statistical analysis, the data were normally distributed and ANOVA was applied. Tukey’s HSD test was used for post-hoc comparisons. Statistical analyses were performed with the Statistica Software (StatSoft Inc). The significance level was P < 0.05.

## Results

Forty one participants were included in the present study: 11 were participants of MA, from which 10 were female, and the average age was 29.5 (±5.8) years; 15 were participants of MwA, from which 14 were female, and the average age was 29.5 (±6.3) years; 15 participants of CG, from which 14 were female, and the average age was 29.1 (±5.0) years (as shown in Table 1).

**Table 1:** Demographic and clinical characteristics of the groups

***Demographic and clinical characteristics of the studied groups***	***Migraine with aura (n = 11)***	***Migraine without aura (n = 15)***	***Controls (n = 15)***	***p-value ANOVA***
**Male/female**	10/1	14/1	14/1	
**Scholarity (years)**	14.5 (±2.8)	14.9 (±2.1)	14.8 (±2.2)	0.8
**Age (years)**	29.3 (±5.8)	29.5 (±6.3)	29.1 (±5.0)	0.9
**Positive family history of migraine**	72.7%	86.7%		
**Attacks frequency (number per month)**	8.2 (±4.1)	7.5 (±2.3)		0.6
**Duration of illness (years)**	17.4 (±7.1)	12.5 (±6.9)		0.08

Data presented as mean (SD) for continuos variables (age, scholarity, attacks frequency and duration of illness) and n (%) for categorical variables (positive family history of migraine).

DPT test results showed a statistically significant difference between the control group and both migraine groups (p<.001). No difference has been observed between the groups with migraine (p = 0.131).

**Figure 1:**
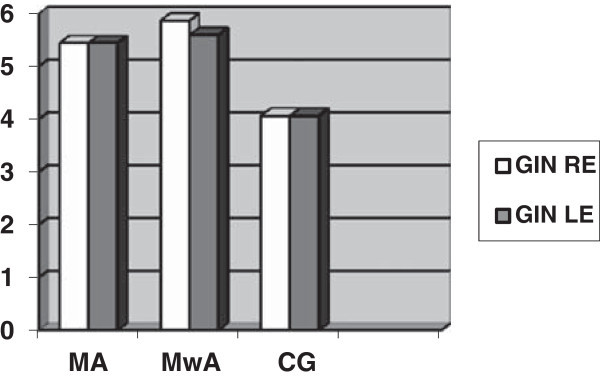
Mean values for the Gaps-in-noise test in right (RE) and left (LE) ears in all groups.

**Figure 2:**
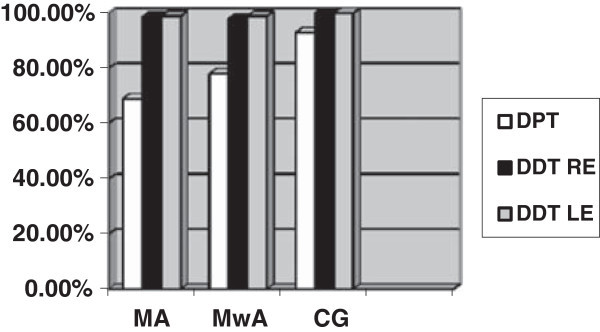
Mean values for DPT and DDT for right (RE) and left (LE) ears in all groups.

The MA had an inferior performance on the GIN test for the left ear (p = .029) and the DPT test for both ears (p<.001). The MwA had a worse performance on the GIN test for the right ear (p = .005) and the left ear (p = .008) and on the DPT test for both ears (p = .003), when compared with the CG. There was no difference between migraineurs and controls on the DDT test (for the right ear p = .362; for the left ear p = .190) and between migraine groups in all tests (as observed in Table 2, Figures 1 and 2).

**Table 2:** Results of the central auditory processing tests of patients with migraine with aura, patients without aura and the controls

**Auditory processing test**	**Migraine with aura group**					**Migraine without aura group**					**Control group**					
	**Ave**	**SD**	**Min**	**Max**	**Med**	**Average**	**SD**	**Min**	**Max**	**Med**	**Average**	**SD**	***Min***	***Max***	***Med***	***p*-value**
**Gaps-in-Noise, RE**	**5.45**	**1.13**	**4**	**8**	**5**	**5.87**	**2.1**	**2**	**10**	**5**	**4.07**	**0.8**	**3**	**5**	**4**	**0.006**
**Gaps-in-Noise, LE**	**5.45**	**1.13**	**4**	**8**	**5**	**5.6**	**1.76**	**2**	**10**	**5**	**4.07**	**0.8**	**3**	**5**	**4**	**0.005**
**Duration pattern test**	**68.90%**	**11.80%**	**56.0%**	**84.0%**	**63.0%**	**77.90%**	**14.60%**	**46.0%**	**100%**	**80%**	**92.80%**	**6.70%**	**76.0%**	**96.5%**	**96.4%**	**<0.001**
**Dichotic digit test, RE**	98.50%	2.30%	92.5%	100%	100%	97.90%	6.10%	76.3%	100%	100%	99.90%	0.30%	98.8%	100%	100%	0.362
**Dichotic digit test, LE**	98.90%	1.80%	95%	100%	100%	98.80%	2.60%	90%	100%	100%	99.90%	0.30%	98.8%	100%	100%	0.190

*Abbreviations: LE* left ear, *RE* right ear, *SD* standard deviation, *Min* minimum, *Max* maximum, *Med* Median. Significant p values (p <0.05) are printed in bold.

## Discussion

The present study demonstrated that individuals with migraine had worse performance in some central auditory processing tests when compared with controls.

The GIN test was performed to assess the auditory ability of temporal resolution (which determines the detection threshold gap or very short silent intervals and is identified as an interruption of the sound stimulus) and the physiological mechanism of temporal processing [26]. Previous research observed, healthy subjects aged between 13 and 46 were evaluated and gap thresholds of 4.8 ms for the left ear and 4.9 ms for the right ear were observed [23]. A hundred young healthy adults aged between 18 and 31 years and obtained threshold gaps of 4.19 ms for both ears in a study [27]. A threshold gap of 3.9 ms for 10 adults was observed [28]. The results of the present study, based on our CG, approached the results found in the literature. A comparison of the descriptive measures showed that migraineurs had similar performance. Comparing groups of migraine patients and the control group showed different performance amongst the groups, to the detriment of both migraineur group. Previous studies have revealed that the GIN test is sensitive for confirming lesions in the central auditory nervous system [23]. The threshold gap of 18 individuals suffering with Parkinson’s disease was evaluated, which showed a deficit of temporal resolution in these patients and hypothesised that a dysfunction exists in the cerebral cortex, especially in the auditory area [29]. The difference in performance on the GIN test between patients with migraine and the control group may denote a central auditory system dysfunction in migraineurs with and without aura. The temporal resolution is essential for speech perception [30].

The DPT test was designed to evaluate the auditory ability of temporal ordering and the physiological mechanism of temporal processing. The normal Brazilian average percentage of correct answers was 83% in a study, that used stimuli with the duration of 250 milliseconds and 500 milliseconds in the ears [31].

The perception of sound stimuli of up to 500 milliseconds involves a basic perceptual sensory mechanism [32]. The neuroanatomical substrate of sound duration discrimination of approximately 300 ms comprises 2 neural networks: a cortical frontal-parietal area (which is responsible for attentional focus to sensory stimuli) and areas involving the basal ganglia, cerebellum, and right prefrontal cortex which are more specifically related to temporal aspects of sound duration discrimination [33]. The DPT detects cortex and inter-hemispheric dysfunctions [34].

In the present study, the average percentages of correct answers from the DPT test were below normal for the groups with migraine—especially for the MA—and within the normal range for the CG. And we therefore observed perceptual impairment in basic sensory processing in migraineurs with and without aura.

The DDT is used to evaluate the figure-ground ability for verbal sounds by using dichotic listening/binaural integration. For an ordinary performance in the DDT, it is necessary that information crosses the corpus callosum and reaches the language-dominant hemisphere. Previous studies suggested that abnormal results in both ears indicate changes in the left hemisphere [35].

The results of this study showed no statically significant difference between the groups that were studied and the performance of individuals within the normal range. Based on that, we could infer that the figure-ground ability for verbal sounds was preserved in people with migraine.

Finally, it should be noted that, some previous studies have shown that migraineurs, in the intercritical phase and in the migraine attack, have abnormalities in the function of neural substrates, responsible for different stages of auditory processing [11,13].

This study had some limitations, including, for example, sample size and gender distribution. The highlights were the selection method which excludes critically ill migraine patients - and the presence of a healthy control group. Further investigation, with larger samples, would be necessary to confirm our findings.

## Conclusions

Patients with migraine had an inferior performance in auditory gap detection and in the discrimination of short and long durations, and presented impairment in the physiological mechanism of temporal processing, especially in temporal resolution and temporal ordering when compared with controls. These difficulties could reflect on auditory memory and attention deficits. Migraine could be related to an impaired central auditory processing.

## Abbreviations

et al.: and others (from the Latin, “et alli”); ICHD – 3 beta: “The International Classification of Headache Disorders– third edition “, (2011); UNIFESP: Federal University of São Paulo; GIN: Gaps-in-noise; DPT: Duration Pattern Test; DDT: Dichotic digit test; CAP: Central auditory processing; MA: Migraine with aura group; MwA: Migraine without aura group; CG: Control group.

## Competing interests

The authors declare that they have no competing interests.

## Authors’ contributions

LMA, MD TRV, MD KZD, MD DSC and MD LDP carried out the studies. LMA carried out the audiological evaluation. LDP and KZD supervised the audiological evaluation. LMA and TRV drafted the manuscript. LMA, TRV and LDP participated in the design of the study, performed the statistical analysis and discussed the data interpretation. TRV, KZD, DSC and LDP reviewed the manuscript and provided useful advice. All authors read and approved the final manuscript.
